# Tunable Magnetism of Organometallic Nanoclusters by Graphene Oxide On-Surface Chemistry

**DOI:** 10.1038/s41598-019-50433-4

**Published:** 2019-10-10

**Authors:** Makoto Sakurai, Pradyot Koley, Masakazu Aono

**Affiliations:** WPI-Center for Materials Nanoarchitectonics (MANA), National Institute for Materials Science (NIMS), Tsukuba, 305-0044 Japan

**Keywords:** Magnetic materials, Magnetic properties and materials, Magnetic properties and materials

## Abstract

Assembly of interacting molecular spins is an attractive candidate for spintronic and quantum computing devices. Here, we report on-surface chemical assembly of aminoferrocene molecules on a graphene oxide (GO) sheet and their magnetic properties. On the GO surface, organometallic molecules having individual spins through charge transfer between the molecule and the sheet are arranged in nanoclusters having diameters of about 2 nm. The synthetic fine tuning of the reaction time enables to change the interspacing between the nanoclusters, keeping their size intact. Their magnetism changes from paramagnetic behavior to collective one gradually as the interspacing decreases. The creation of collective nature among weakly interacting molecular spins through their nanoscale arrangement on the GO surface opens a new avenue to molecular magnetism.

## Introduction

Assembly of interacting molecules is promising in the field of molecular spintronics because of its small size, functionality created by their assembling, and diversity of their combinations. Single-molecule-magnet (SMMs)^1^ is one of the most important building blocks in the field, and their aggregation enables to tune magnetic properties of an isolated one^2^. The connection of SMMs has been designed and built up for the achievement of quantum computation devices^3,4^. For networking assembly of SMMs on a surface, their chemical, structural, and magnetic features have been characterized by using several techniques^5^. Recently, the interactions between clusters formed by SMMs and a graphene substrate have been reported to cause the modification of magnetic relaxation through phonon excitations in graphene^6^. Here, we have developed different approach to make assemblies of weakly interacting molecular spins by using on-surface chemical synthesis. We have fabricated organometallic nanoclusters with average diameter of about 2 nm on a graphene oxide (GO) surface. We studied magnetic properties of the resulting nanoclusters by changing reaction time of the synthesis to show how nanoscale arrangement of the nanoclusters leads to the appearance of collectivity among the spins. We also compared the collective behaviors among spins in aminoferrocene functionalized GO (AFc-GO) sheets with those in spin glasses^7–9^, single-chain magnets^10–12^, and ferromagnetic nanoparticles^13–18^.

GO with organometallic aminoferrocene in an *N*,*N*-dimethylformamide solution was functionalized by using a technique initially developed for covalent immobilization of proteins on carboxylated carbon nanotubes (CNT)^19^. Here, covalent immobilization between the carboxyl groups of the GO sheet and the amino groups of aminoferrocene molecule is promoted by coupling agents (see Methods). After the mixtures were stirred for a certain time (reaction time) to cause the reaction, they were separated and subsequently purified to produce AFc-GO sheets. The surface morphology of AFc-GO sheets were modulated by tuning the reaction time.

**Figure 1:**
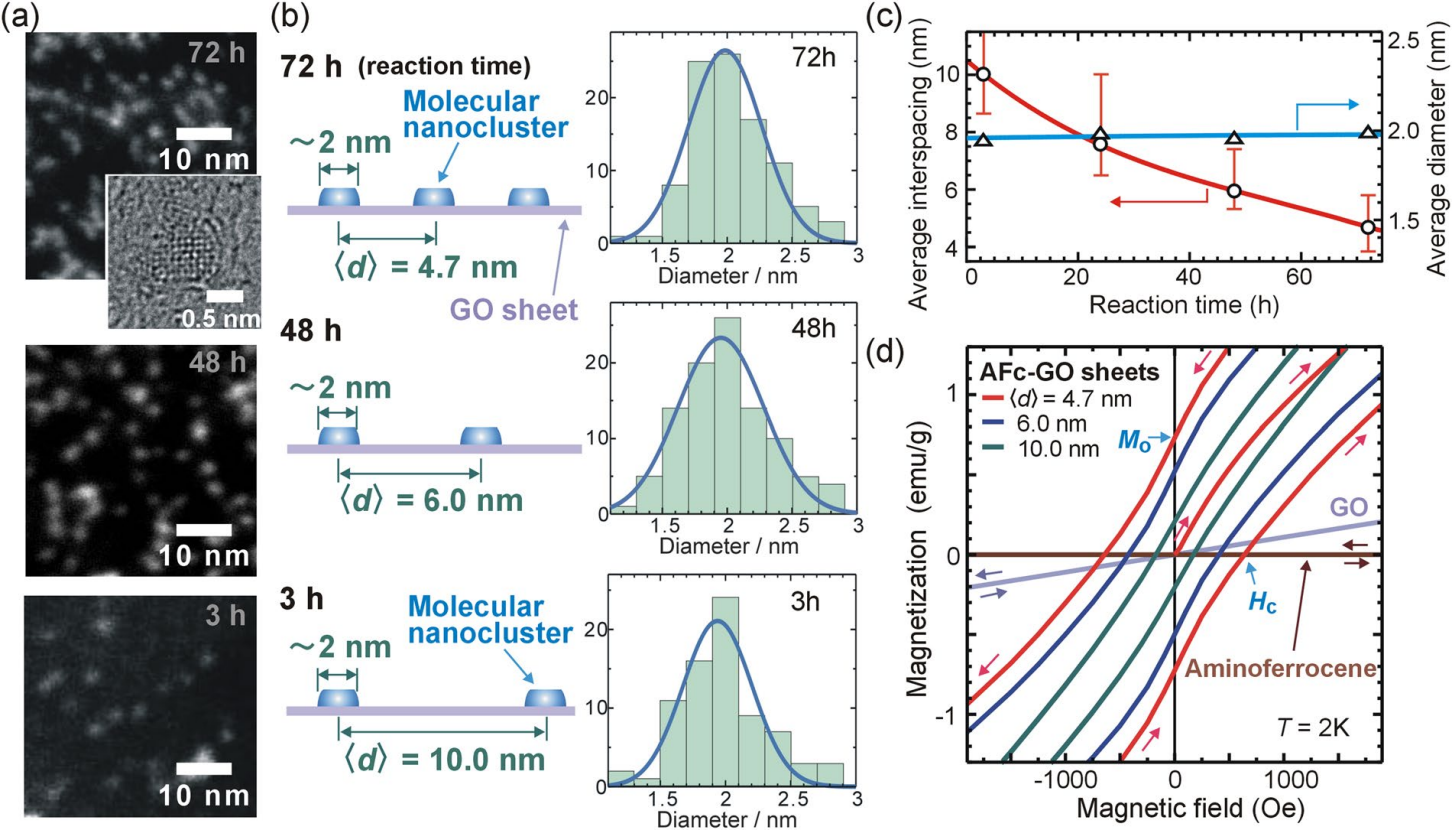
(**a**) Scanning transmission electron microscope (STEM) images of GO sheet functionalized by aminoferrocene (AFc-GO) taken after 3, 48, and 72 h of synthesis. Inset: High-resolution TEM image of a molecular nanocluster after 72 h synthesis. (**b**) Schematic illustration of nanoclusters on a GO sheet, average interspacing between the nanoclusters, and histogram of their diameters for each reaction time. (**c**) Average interspacing between the nanoclusters and average diameter of nanoclusters versus reaction time of synthesis. (**d**) *M*-*H* loop near *H* = 0 Oe for AFc-GO sheets for each reaction time, pristine GO sheets, and aminoferrocene molecules at *T* = 2 K. Note that the initial magnetization curve from the demagnetized state has been added to the loop of the AFc-GO sheets (72 h synthesis, $\langle d\rangle$ = 4.7 nm).

The functionalized GO sheets were characterized using transmission electron microscopy, X-ray photoelectron spectroscopy (XPS), Fourier transform infrared spectroscopy (FT-IR), and mass spectroscopy. The scanning transmission electron microscope (STEM) image of an AFc-GO sheet taken after 72 h of synthesis displays bright spots (Fig. 1a) with an average diameter of ~2.0 nm (Fig. 1b), average number density of 4.6 × 10^−2^/nm^2^, and average height of ~1.5 nm (Supplementary Fig. S1). As the reaction time increased, the number density of the nanoclusters increased (Fig. 1a,b, Supplementary Fig. S2a). Energy dispersive X-ray (EDX) analysis clearly showed that the Fe components were associated with the bright spots (Supplementary Fig. S3). The FT-IR spectra of the AFc-GO sheets (Supplementary Fig. S4) were characterized by the peaks corresponding to GO^20–22^ as well as the peaks corresponding to ferrocene^20,23,24^ or aminoferrocene^25^ molecules. The electronic states of the atoms forming the sheets (Supplementary Fig. S5a), as monitored through XPS, were found to change at each stage of the synthesis (Supplementary Figs S5b and S6a). The main peak in the C1s core level of the sheets changed from that of pristine GO (~286.5 eV) to that of aminoferrocene (~284.5 eV) as the synthesis progressed, which is consistent with the increase in the number density of the nanoclusters on the GO sheets (Supplementary Fig. S2a). The ionized states of Fe were ascertained from the position of the peak of the Fe 2p core level, because the position of this peak is very sensitive to the ionic state^26^. After synthesis, the peak shifted in energy (Supplementary Fig. S6b), suggesting the ionized state changed from Fe^+2^ to Fe^+3^ as a result of electron transfer from the Fe ions to the GO sheets. Such charge transfer between ferrocene or substituted ferrocene and a GO sheet has been reported previously^23–25^, where the sheet played the role of an electron acceptor, while the ferrocene or substituted ferrocene worked as an electron donor. The XPS peak of the N 1 s core level was observed after the synthesis (Supplementary Fig. S7a) and the main peak in N 1 s core level (~399.7 eV) (Supplementary Fig. S7b) was attributed to a nitrogen atom linked to a carbon atom by a single bond^27^, indicating the formation of the covalent bond (see Supplementary Fig. S5c). From the atomic components obtained from XPS signals of AFc-GO sheets (Supplementary Fig. S7b–d), the Fe/N ratio was close to 1 and the Fe/C ratio increased (Supplementary Fig. S7e) as the synthesis progressed. Peak at 201 amu in the mass spectrum of AFc-GO sheets (Supplementary Fig. S8) corresponds to aminoferrocene molecules (Fe(C_5_H_5_)_2_NH_2_) with atomic weight of 201.05 amu or ionized molecules (Fe(C_5_H_5_)_2_NH^+^). From these results we concluded that the nanoclusters were composed of aminoferrocene molecules or its ionized ones.

The distribution, arrangement, and structure of the nanoclusters on a GO sheet are further analyzed by using their TEM images. The average interspacing between the nanoclusters (Fig. 1c) and number of reacted molecules (Supplementary Fig. S2b) changed as functions of the reaction time. The distribution of the nanoclusters on the surface was estimated using a quadrat counting method (Supplementary Fig. S2c) and spatial point analysis (see Supplementary discussion and Supplementary Fig. S9); the results suggested that nanoclusters distributed almost uniformly on the surface without forming large aggregations. Note that the syntheses produced nanoclusters with the same average diameters (Fig. 1c). Hereinafter, we will use the average interspacing between the nanoclusters to distinguish the samples; the conversion relation is indicated by the red curve in Fig. 1c. The lateral arrangement of molecules within the nanocluster on a GO surface was estimated by using Fourier transform analysis to highly resolved TEM images (Supplementary Fig. S10). It revealed a square symmetry with a periodicity of ~0.33 nm, suggesting dense and regular arrangements of molecules laterally (see schematic drawing in Supplementary Fig. S10f,g). It differs from that of aminoferrocene crystals^28^.

The growth of the nanoclusters on the surface proceeded by diffusion of molecules or atoms on the surface and adsorption at nucleation sites^29–31^. The fact that there were no nanoclusters at the edges of the GO sheets in the TEM images (Supplementary Fig. S11) indicated a role of surface diffusion on the growth. Here, a nucleation site could have arisen from an aminoferrocene molecule covalently bonded with the GO sheet through the coupling reaction (Supplementary Fig. S5c). There are two explanations for the uniform size of the nanoclusters through the on-surface chemical synthesis. One is based on the microscopic balance between the interface energy and the volume energy of the nanocluster on the sheet, similar to the formation of a droplet on a surface^30^. The other is based on the limitation of the charge transfer between the molecule and GO sheet. As the size of the nanocluster increases, the path of the charge transfer through the covalent bond of the molecule bonding to the sheet becomes longer and the difficulty of the transfer increases exponentially^32^.

The functionalization of the GO sheets by aminoferrocene enhanced their magnetization greatly. The magnetizations at a magnetic field *H* of 70 kOe were 9.2 emu/g at *T* = 2 K for the AFc-GO sheets (average interspacing $\langle d\rangle$ of 4.7 nm), 1.5 emu/g for the GO sheets, and 2 × 10^−2^ emu/g for the aminoferrocene molecules (Supplementary Fig. S12a). This value is consistent with the results of the XPS spectra; the peak of the Fe 2p core level for the pristine aminoferrocene molecules was mainly due to Fe^2+^ ions in the molecule (top panel in Supplementary Fig. S6b), whose energy levels of the *d*-orbitals were split by a ligand field^33^, indicating a zero spin state. The peak of Fe 2p core level for the AFc-GO sheets was mainly due to Fe^3+^ ions (Supplementary Fig. S6b), and it corresponded to a spin state (*S* = 1/2)^33^. The chemical stability of ferrocene or aminoferrocene molecules suggests the exclusion of a possibility forming oxidized Fe nanoclusters in the synthesis. Since a strong magnetic field (*H* > 70 kOe) is needed for saturation of the magnetization for AFc-GO sheets (Supplementary Fig. S12a), then the result also supports the exclusion of oxidized Fe nanoclusters, because larger magnetic moments of the ferromagnetic nanoclusters lead to much weaker field (*H* ~ 1 kOe) for their saturation.

The magnetic moment *m*_AFc_ of an aminoferrocene molecule forming the nanoclusters is estimated using the saturated magnetization *M*_tot_ of the AFc-GO sheets. The relationship between *M*_tot_ and *m*_AFc_ is expressed as

1 $${M}_{{\rm{tot}}}=\frac{{M}_{{\rm{GO}}}\times {W}_{{\rm{GO}}}+{m}_{{\rm{AFc}}}\times {N}_{{\rm{NC}}}\times {D}_{{\rm{NC}}}}{{W}_{{\rm{GO}}}+{W}_{{\rm{AFc}}}\times {N}_{{\rm{NC}}}\times {D}_{{\rm{NC}}}}$$

here, *M*_GO_ is the magnetization of the GO sheets, *W*_GO_ is the weight of the GO sheet per unit area, *W*_AFc_ is the weight of a single aminoferrocene molecule, *N*_NC_ is the number of molecules within the nanocluster, and *D*_NC_ is the number density of nanoclusters on the GO sheet. If we take *M*_tot_ to be magnetization (9.2 emu/g) of AFc-GO sheets (*d* = 4.7 nm) at *H* = 70 kOe and *T* = 2 K and *N*_*NC*_ to be 100, which is roughly estimated from the average size of the nanocluster (Supplementary Figs S1c and S10g), Eq. (1) gives an *m*_AFc_ of 0.4 *μ*_*B*_. This value was smaller than the 1 *μ*_*B*_ of the ionized Fe atoms (Fe^+3^) estimated from the XPS spectra of the Fe 2p core level (Supplementary Figs S6b and S7c). This discrepancy was caused by the fact that we underestimated the saturated magnetization of the AFc-GO sheets. The magnetization in the *M*-*H* curve of the AFc-GO sheets did not saturate at *H* = 70 kOe (Supplementary Fig. S12a). A lot of water was included in the sample because of the hydrophilicity of the sheet. Therefore, the magnetic moment per molecule within the nanocluster would be more close to 1 *μ*_*B*_. This in turn means that we produce a novel molecular spin system where about 100 molecules having individual spins are arranged within nanoclusters with a diameter of about 2 nm, whose interspacing on the GO surface is adjustable by synthetic tuning of the reaction time.

Hysteresis characterized by a coercive force *H*_c_ was clearly observed in the magnetization curves of the AFc-GO sheets (Fig. 1d). The *H*_c_ of the AFc-GO sheets increased as the average interspacing between the nanoclusters decreased (Supplementary Fig. S12b,c), suggesting the increase in the resistive nature against spin inversion. For the GO sheets, in contrast, the curve changed linearly, displaying no hysteresis (Fig. 1d).

**Figure 2:**
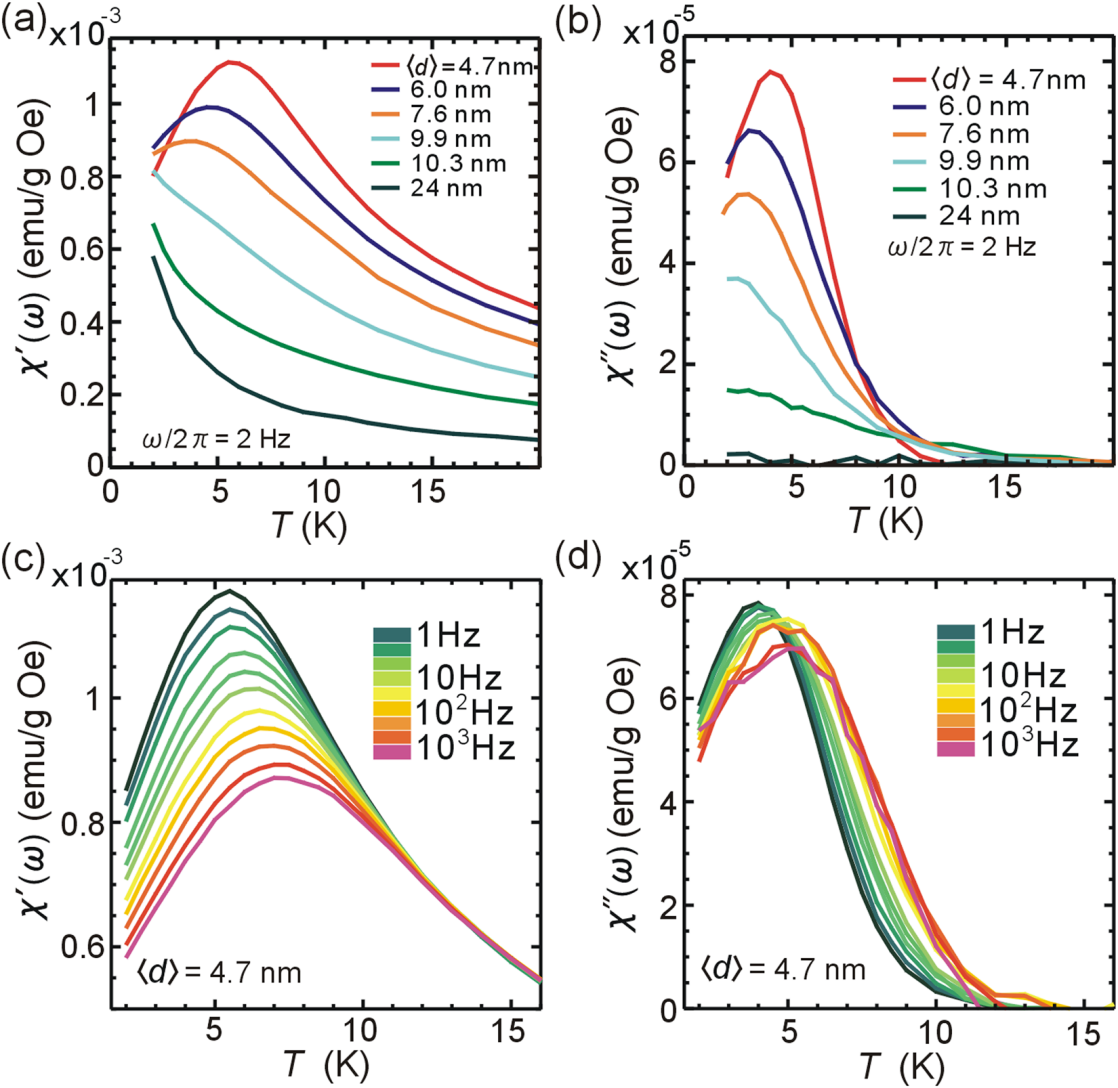
(**a**) Real component *χ*′ (ω) and (**b**) imaginary component *χ*″ (ω) of the dynamic magnetic susceptibility of AFc-GO sheets ($\langle d\rangle$= 4,7, 6.0, 7.6, 9.9, 10.3, and 24 nm) with a frequency of 2 Hz in a zero static magnetic field are plotted against temperature. (**c**) *χ*′ (ω) and (**d**) *χ*″ (ω) of AFc-GO sheets ($\langle d\rangle$= 4.7 nm) in a zero-field plotted against temperature.

The dynamics of AFc-GO sheets was probed by using an AC SQUID (superconducting quantum interference device) magnetometer to measure the real *χ*′ (ω) and imaginary *χ* (ω) responses to a small magnetic field oscillating at a frequency ω/2π without applying a static magnetic field (Fig. 2a,b). The AFc-GO sheets whose average nanocluster interspacing was about 24 nm (Supplementary Fig. S13a) showed paramagnetic behaviors, i.e., no hysteresis in the *M*-*H* loop (Supplementary Fig. S13b), a monotonic change in *χ*′ (ω) against temperature (Fig. 2a, Supplementary Fig. S13c), and no *χ*″ (ω) (Fig. 2b, Supplementary Fig. S13d). The paramagnetic nature of the nearly isolated nanoclusters is attributed to weak exchange interactions between the molecular spins and to the large surface-to-volume ratio. The magnetic anisotropy in the nearly isolated nanoclusters was negligibly small compared with thermal energy (*T* ≥ 2 K). As the average interspacing between the nanoclusters was decreased, the monotonic *χ*′ (ω) curves changed into complex ones (Fig. 2a,b), suggesting that the paramagnetic nature of the nanoclusters gradually became a collective one. For the AFc-GO sheet with $\langle d\rangle$ ≤ 7.6 nm, the dynamic susceptibility increased as the temperature decreased; moreover, it reached a maximum in the low temperature range, *T* < 6 K. The emergence of *χ*″ (ω) indicates the onset of energy dissipation and spin correlations. As the interspacing decreased, the effect of spin correlations: *χ*″ (ω) (Fig. 2b) increased.

The relationship between the change in dynamic response and the arrangement of nanoclusters on the surface was investigated by measuring frequency-dependence of *χ* (ω). The *χ*′ (ω) and *χ*″ (ω) peaks of the AFc-GO sheets ($\langle d\rangle$ = 4.7 nm) shifted to higher temperature and the intensity of both susceptibilities decreased, as the driving frequency was increased (Fig. 2c,d). The frequency-dependent features indicate blocking of oscillatory spins, which has been reported in spin glasses^7–9^, single-chain magnets^10^, and ferromagnetic nanoparticles^15^. *T*_p_(*χ*′) was the peak temperature giving the maximum in *χ*′ (ω) for AFc-GO sheets (*d* ≤ 7.6 nm), which was determined by Gaussian fitting to the data (Supplementary Fig. S14a). The inverse of *T*_p_(*χ*′) was proportional to ln(*ω*/2*π*) (Supplementary Fig. S14b), indicating that the blocking followed a thermal activation process known as the Arrhenius law^7,8^: *ω*/2*π* = *f*_0_exp(−*E*_a_/*k*_B_*T*_p_(*χ*′)), where *E*_a_ is the energy of the blocking and *f*_0_ is a pre-exponential factor. Parameters *E*_a_/*k*_B_ and *f*_0_ obtained from a linear fitting to the data for the AFc-GO sheets were 161 K and 4 × 10^−13^ s for ⟨*d*⟩ = 4.7 nm, 117 K and 7 × 10^−12^ s for ⟨*d*⟩ = 6.0 nm, and 96 K and 4 × 10^−12^ s for $\langle d\rangle$ = 7.6 nm. The blocking energy increased as the interspacing between the nanoclusters decreased.

Dynamic response of AFc-GO sheets was compared with that of spin glass materials. The dynamic susceptibilities of the AFc-GO sheets (⟨*d*⟩ = 4.7 nm) at fixed temperature (*T* = 2, 4, 6, and 8 K) are plotted in Fig. S15 against the driving frequency. The dynamic response of AFc-GO sheets has following features: *χ*″ (ω) is more than one order of magnitude smaller than *χ*′ (ω); both *χ*′ (ω) and *χ*″ (ω) depend weakly on the frequency. Such features make difficult to draw a semicircle using *χ*′ (ω) and *χ*″ (ω) of AFc-GO sheets in the Cole-Cole plot^1^ (Supplementary Fig. S16). The property is different from that of single-chain magnets^10,11^ or ferromagnetic nanoparticles^16,17^, where their dynamic susceptibilities (*χ*′ (ω), *χ*″ (ω)) form a clear semicircle in the Cole-Cole plot. Frequency shift obtained from Γ = (1/*T*_*p*_)Δ*T*_*p*_/Δ(*log*_10_(*ω*/2*π*))^8,9^ was calculated for AFc-GO sheets: *Γ* = 0.12 (⟨*d*⟩ = 4.7 nm), 0.14 (⟨*d*⟩ = 6.0 nm), and 0.13 (⟨*d*⟩ = 7.6 nm). The values were larger than those of spin glasses (*Γ* = 0.0045–0.06)^9^ and were close to that of single-chain magnets (*Γ* ≈ 0.13)^11^ or ferromagnetic nanoparticles (*Γ* = 0.1–0.3)^8,9^. Since molecular spins of AFc-GO sheets have weak exchange interaction and negligibly small uniaxial anisotropy, then the mechanism of the frequency-dependent phenomena for AFc-GO sheets should be different from that for single-chain magnets^11,12^ or ferromagnetic nanoparticles^15,18^.

**Figure 3:**
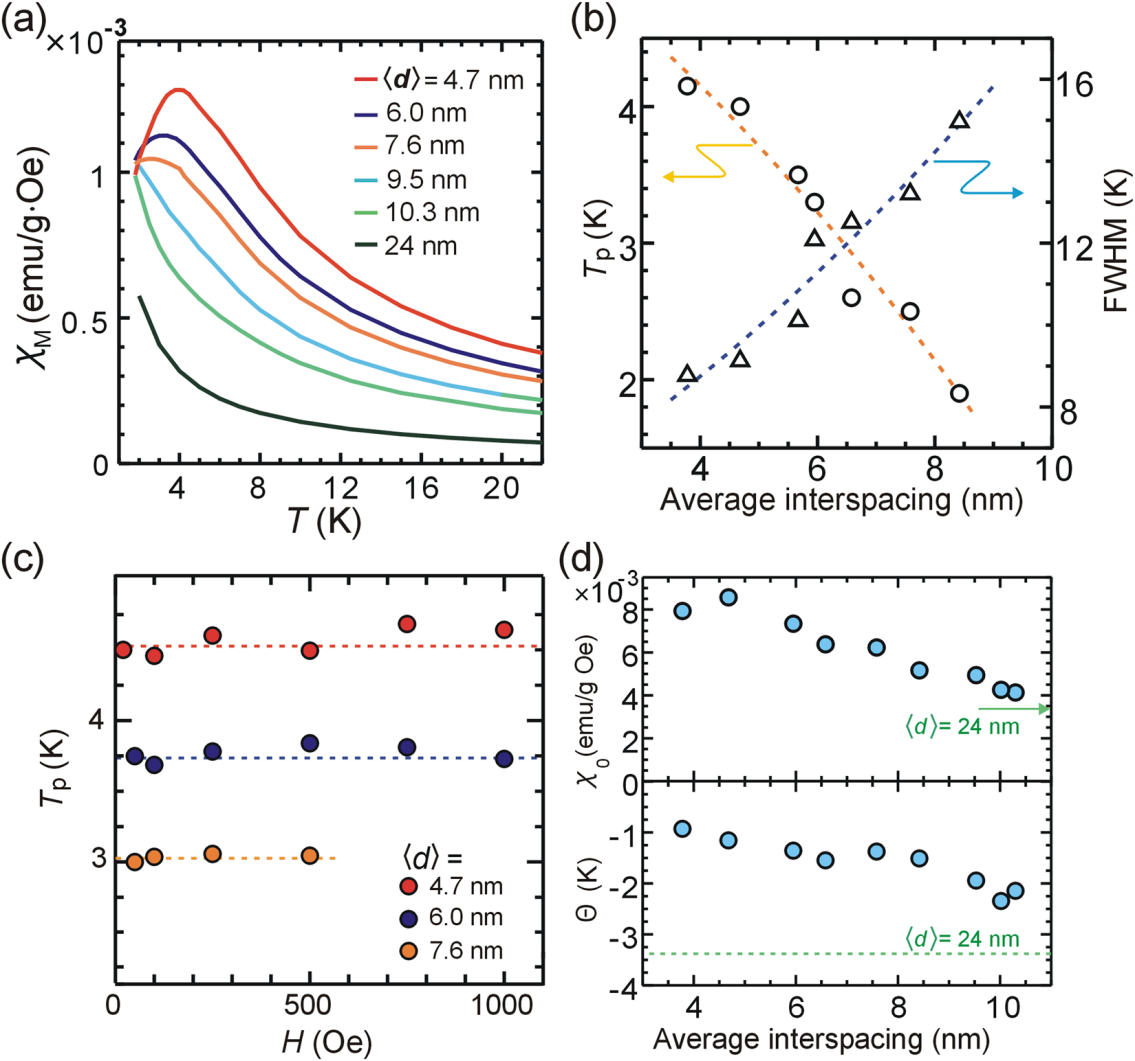
(**a**) Static magnetic susceptibility *χ*_M_ = *M*/*H* using magnetization M at *H* = 500 Oe in the initial curve of AFc-GO sheets as a function of temperature. (**b**) Peak temperature *T*_p_ and full width at half maximum (FWHM) to the *χ*_M_ peak are plotted against the average interspacing between the nanoclusters. The dashed line is a guide to the eye. (**c**) Temperature *T*_p_ for *χ*_M_ peak under a static field for AFc-GO sheets ($\langle d\rangle$ = 4.7, 6.0, 7.6 nm) is plotted against the field. The dotted line in (**c**) corresponds to averaged value for each sheet. (**d**) Parameters *χ*_0_ and *Θ* of AFc-GO sheets are plotted against average interspacing between the nanoclusters. Parameters *χ*_0_ and *Θ* of AFc-GO sheets (〈d〉 = 24 nm) are marked by green arrow and dotted line, respectively.

Peak shift and broadening of full width at half maximum (FWHM) in static susceptibility *χ*_M_ = *M*/*H* of the AFc-GO sheets (Fig. 3a) depended on the average interspacing between the nanoclusters (Fig. 3b). Since the peak temperature *T*_p_ is determined by the energy balance between the blocking and thermal activation, the decrease in *T*_p_ suggests a weakening of the blocking. The broadening of the FWHM is due to the decrease in the spin correlations. As the interspacing decreased, the resistive behaviors such as spin correlation and blocking decreased, similar to the results in *χ*″ (ω) (Fig. 2b) and *H*_c_ (Supplementary Fig. S12b).

Magnetic anisotropy of AFc-GO sheets is estimated through the measurement of *χ*_*M*_ under the application of a static magnetic field (Supplementary Fig. S17). The peak temperature *T*_p_ for the *χ*_M_ peak of AFc-GO sheets (⟨*d*⟩ = 4.7, 6.0, 7.6 nm) was independent of the intensity of the applied field (Fig. 3c), suggesting that there is negligibly small uniaxial anisotropy from a following reason. For spin systems with uniaxial anisotropy (e.g. ferromagnetic nanoparticles), spins are forced to align in the direction of the uniaxial anisotropy. The applied field modifies barrier height formed by the anisotropy^1,18^, leading to a change of the blocking and a shift of *T*_p_.

The magnetic interactions operating to the nanoclusters for AFc-GO sheets are estimated by using the Curie-Weiss law: *χ*_M_ = *χ*_0_/(*T*−Θ)^34,35^ in the high temperature region (*T* > 15 K). From the linear fitting to *χ*_M_^−1^ (Supplementary Fig. S18), we obtained *χ*_0_ and *Θ* for the AFc-GO sheets as a function of the average interspacing between the nanoclusters (Fig. 3d). In this figure, the monotonic feature of *χ*_0_ versus the interspacing is mainly due to the change in the number density of the nanoclusters (Supplementary Fig. S2a). The nearly isolated nanoclusters (⟨*d*⟩ = 24 nm) had *Θ* of −3.3 K, suggesting that the value is mainly due to the demagnetizing field within the nanoclusters^36^. Since all the nanoclusters have almost the same structures, the same demagnetizing field could be induced in the nanoclusters on AFc-GO sheets. Then the difference between *Θ* and −3.3 K (dotted line in Fig. 3b) is interpreted as magnetic interactions from surrounding nanoclusters. The difference gives a positive value, suggesting that there is a ferromagnetic interaction whose intensity decreases as the interspacing increases. From XPS spectra of the sheet, the C/O ratio calculated dividing the total C 1 s spectral area by the total O 1 s spectral area of the GO sheets was about 1.8 (Supplementary Fig. S7d), suggesting highly oxidation of carbon atoms forming the sheets. Therefore, there are three possible types of magnetic interactions between the molecular spins mediated by the GO sheet: interaction through local conductive region (graphene-like region) on the sheet, super-exchange interaction through the oxygen^32^ bonding to the surface, and interaction through the carbon^32^ forming the sheet. In addition, there are magnetic dipole interactions between the molecular spins. Although it is difficult to estimate the ratio of the possible interactions, the experimental results show that the ferromagnetic interactions from neighboring nanoclusters play an important role in the creation of the resistive nature such as spin correlation, coercive force, and blocking in the nanoclusters.

## Conclusions

We characterized organometallic nanoclusters grown by using GO on-surface chemical synthesis and demonstrated tunable magnetic properties of weakly interacting molecular spin systems through their nanoscale arrangement on the surface. Our measurements revealed a gradual change from a paramagnetic nature to a collective one as the interspacing between the nanoclusters decreased. Even through molecular spins within the nanoclusters had small exchange interactions and negligibly small uniaxial anisotropy, spin correlations and blocking phenomena of the weakly interacting spins became observable gradually by the increase in the ferromagnetic interactions from the neighboring nanoclusters as the interspacing decreased. The novel nature created by arranging weakly interacting molecular spins on a surface paves the way for the design and synthetic fabrication of new platforms for future spintronic and quantum computing devices.

## Methods

### Nanocluster formation

One mL of the water-dispersed GO solution (4 mg/mL) was mixed with 50 mL of DMF in a round bottom flask and cooled in an ice bath followed by the addition of solid aminoferrocene (20 mg, TCI). The coupling reaction between the carboxyl group of graphene oxide and amino group of aminoferrocene (Fig. S5c) was achieved after quantitatively adding coupling agents (EDC-HCl, HOBt and trimethylpyridine) to the ice-cold solution of reaction mixture. The solution was stirred at 0 °C for 3 hours to control any excessive reaction in the initial stage of the synthesis and subsequently stirred at room temperature. To tune the number density of the nanoclusters on the GO sheets, the synthesis was performed for a given time scale (reaction time), and the products were separated quickly from the supernatant liquid by using an ultrafast centrifuge. The as-synthesized products were purified through repeated washing, first with DFM and then with distilled water to remove any unreacted chemicals from the sheets. After drying, AFc-GO sheets were produced that had a tuned average interspacing between nanoclusters. As for the formation of the nanoclusters on a GO sheet (⟨*d⟩* = 24 nm), 14 mg of aminoferrocene was dispersed in the GO solution and the same synthesis was performed for 1.5 h.

### Magnetic measurements

The *M*-*H* loop was measured for aggregations of AFc-Go sheets with a weight of 5–30 mg using a SQUID magnetometer (MPMS-7T, Quantum design). All of the magnetic data were shown without subtracting the background. The static magnetic susceptibility *χ*_*M*_ = *M*/*H *was obtained from the magnetization *M* at a static magnetic field *H* in the initial magnetization curve at each temperature. The dynamic magnetic susceptibility was measured at frequency range between 1–950 Hz in an oscillating field of 0.5–2 Oe using a SQUID magnetometer (MPMS-1T, Quantum design). Although the magnetic measurements were performed macroscopically on aggregations of sheets, the magnetic interactions between nanoclusters on different sheets were negligibly small due to the long distance, because the sheets in the aggregations did not adhere closely as a result of there being wrinkles, folds, and water on the surface.

### Characterization of the nanoclusters

After separation of the as-synthesized products from the solution at each reaction time using an ultrafast centrifuge, the Fe concentration in the unreacted supernatant liquid was measured using inductively coupled plasma optical emission spectroscopy **(**ICP-OES) to estimate the progress of the synthesis reaction (Supplementary Fig. S2b). Molecular components forming the AFc-GO sheets were characterized using FT-IR spectrophotometer (JASCO, V-7200). The electronic states of atoms forming the AFc-GO sheets were characterized using X-ray photoelectron spectroscopy (XPS) spectra (Thermo Fisher Scientific Thera Probe System) of the C1s and Fe 2p core levels for each reaction time. The average interspacing between the nanoclusters was calculated using the number density obtained from the TEM or STEM images.

### TOF-SIMS characterization

TOF-SIMS analysis was performed with a time-of-flight (TOF)-secondary ion mass spectrometer (SIMS) (PHI TRIFT V nanoTOF, Ulvac-Phi) equipped with a primary ion source of Bi_3_^2+^. Positive ion spectra were acquired with a pulsed, bunched 30 kV Bi_3_^2+^ primary ion beam by scanning the ion beam over a 300 μm × 300 μm sample area.

## Supplementary information

Supplementary

## Data Availability

The datasets generated during the current study are available from the corresponding author on reasonable request.
