# Supplementary material for: Tunable Magnetism of Organometallic Nanoclusters by Graphene Oxide On-Surface Chemistry
Source: Sci Rep. 2019 Oct 10;9:14509. doi: 10.1038/s41598-019-50433-4 (PMC6787201; doi:10.1038/s41598-019-50433-4)
Supplement: Supplementary file 1 — Supplementary [file 41598_2019_50433_MOESM1_ESM.pdf]

Supplementary Information

Tunable Magnetism of Organometallic Nanoclusters by Graphene Oxide On-Surface Chemistry

Makoto Sakurai*, Pradyot Koley, and Masakazu Aono

WPI-Center for Materials Nanoarchitectonics (MANA),

National Institute for Materials Science (NIMS), Tsukuba 305-0044, Japan

E-mail: sakurai.makoto@nims.go.jp

Contents

Supplementary Discussion

Spatial point processing for the distribution of nanoclusters

Supplementary References

Supplementary Figures (Figure S1 - Figure S18)

Supplementary Discussion

Spatial point processing for the distribution of nanoclusters: The distribution of nanoclusters on a GO sheet was analyzed using spatial patterns obtained from their TEM images.

The nanoclusters on AFc-GO sheets with different reaction times were drawn as points in Figure S6a. The point patterns were analyzed using the K-function: [1,2]

$K(h)$ = (number of dots within distance h from a point) / λ. (S1)

Here, λ is average number density. Figure S6b plots $K(h)$ against distance h for each AFc-Go sheet. To remove the edge correction in the statistical processing, we did not include the points in the area within 10 nm of the boundary of the map. The deviation from the line at $h < \sim 2$ nm is due to that other nanoclusters don't form within ~2 nm because of the finite size of the nanoclusters. $K(h)$ for each AFc-GO sheet has a similar tendency as the solid curve assumed a uniform distribution, suggesting that there are no large aggregations of nanoclusters. The average number density at a distance h for each point in the map is plotted in Figure S6c, and its distribution is close to the average value (red dotted line).

Supplementary References

(1) Ripley, B. D. *Statistical inference for spatial process*, Cambridge Univ. press, Chap. 2 (1991).

(2) Møller, J.; Waagepetersen, R. P. *Statistical inference and simulation for spatial point processes*, Chapman & Hall/CRC, Chap. 4 (2004).

(3) Halland, A. Molecular structure and bonding in the 3d metallocenes. *Acc. Chem. Res.*, **12**, 415-422, (1979).

(4) Barazzouk, S.; Daneault, C. Amino Acid and Peptide Immobilization on Oxidized Nanocellulose : Spectroscopic Characterization, *Nanomaterials* **2**, 187-205 (2012).

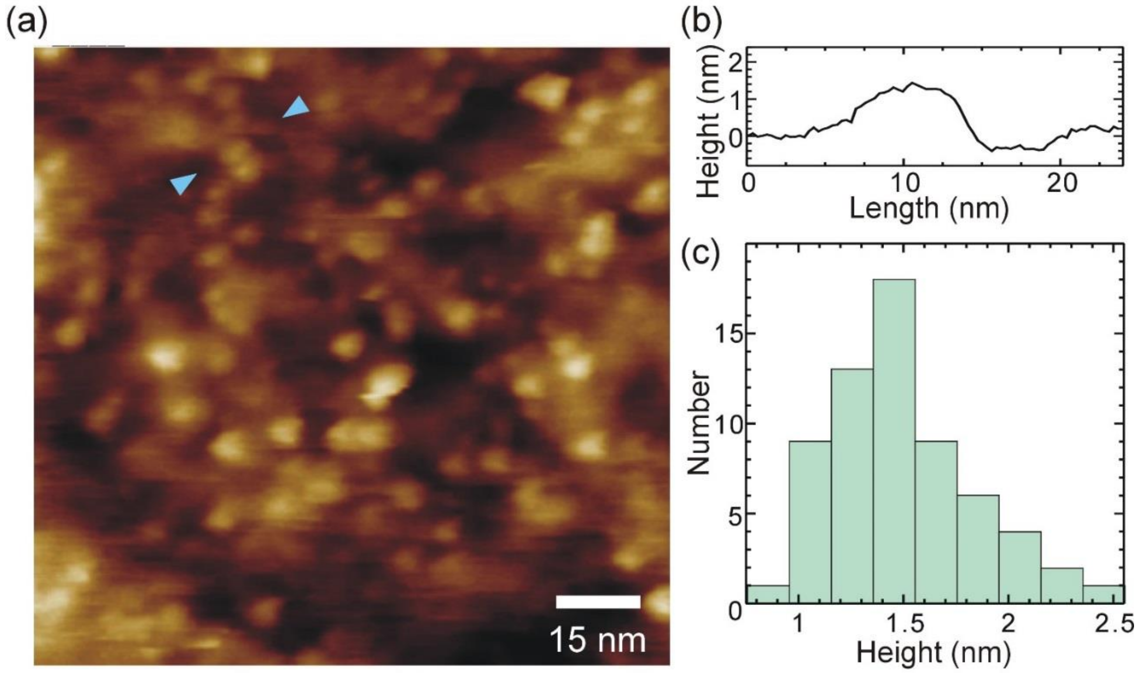

Figure S1. (a) Atomic force microscope (AFM) topological image of an AFc-GO sheet after a reaction time of 72 h. (b) Cross-sectional line profile of the nanocluster marked by blue arrows in (a). (c) AFM height distribution of the nanoclusters after a reaction of 72 h on a graphene oxide sheet, indicating an average height of 1.5 nm.

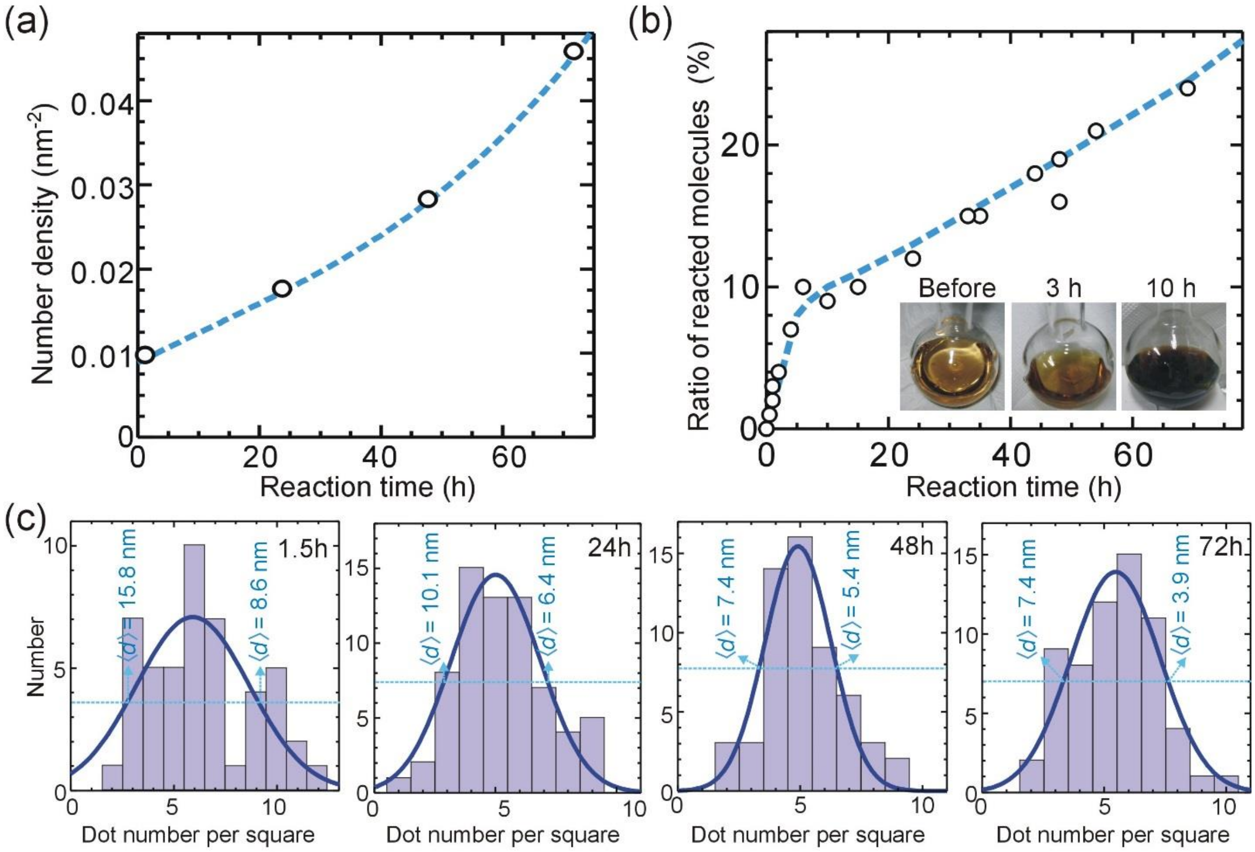

Figure S2. (a) Number density of the nanoclusters on the GO sheets synthesized at each reaction time, which were estimated from their TEM images. (b) The ratio of the reacted aminoferrocene molecules to the molecules in the solution before reaction is plotted against the reaction time. The difference between the Fe concentration of the original solution and the Fe concentration in the supernatant liquid separated from the solution at a reaction time, measured by using ICP-OED, corresponds to the Fe concentration adsorbed on a GO sheet at the reaction time. The increase of the ratio means progress of the reaction forming the molecular nanoclusters. Inset: Pictures of round bottom flasks before and after reaction times of 3 h and 10 h. (c) The histogram created using the quadrat count method shows the number of nanoclusters in a small square of 25.8 × 25.8 nm^2 (1.5 h), 17.2 ×17.2 nm^2 (24 h), 13.6 × 13.6 nm^2 (48 h), 10.6 ×10.6 nm^2 (72 h). The width of the Gaussian fitting curve gives the deviation of the average interspacing between the nanoclusters, which is shown as the error bar in Figure 1c.

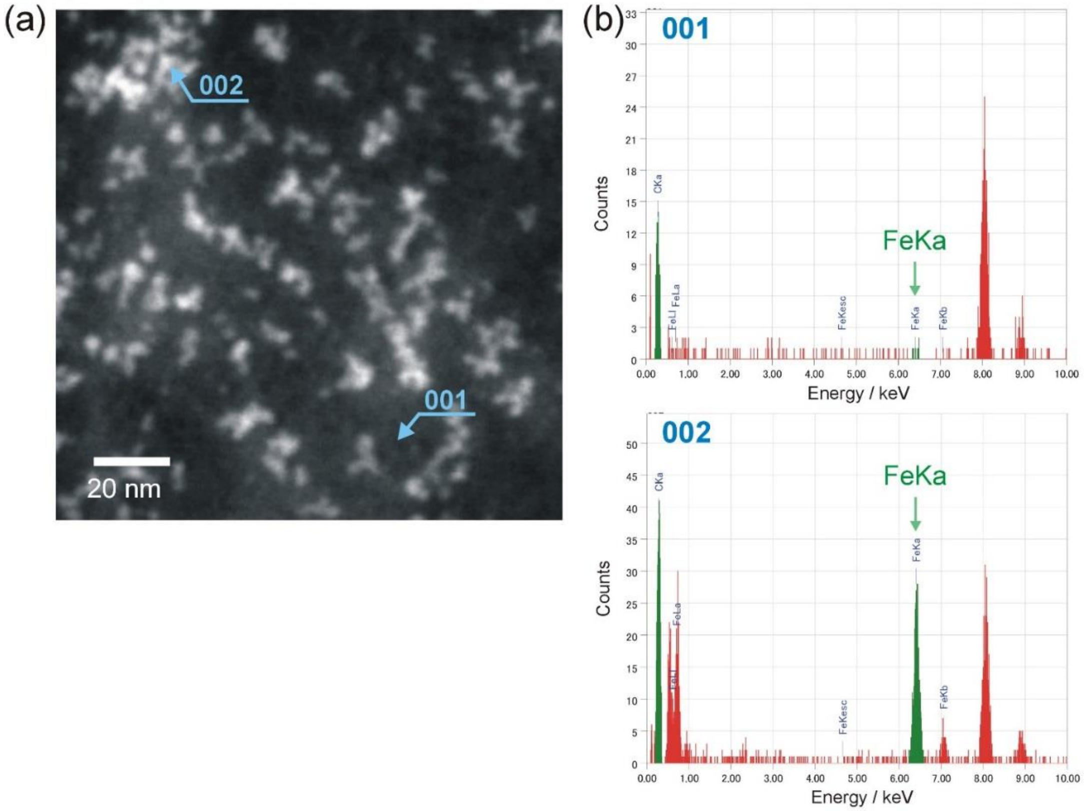

Figure S3. (a) Scanning transmission electron microscope (STEM) image of an AFc-GO sheet after a reaction time of 72 h. (b) Local EDX spectra measured in each area as marked in (a). Fe signals are observed only on the nanoclusters, suggesting that the nanoclusters formed from aminoferrocene molecules.

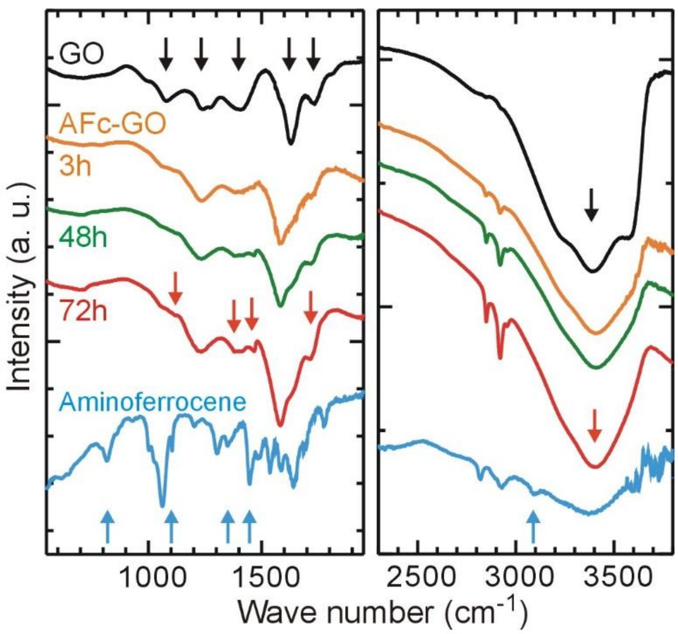

Figure S4. FT-IR spectra of GO sheets, AFc-GO sheets (reaction time: 3 h, 48 h, 72 h), and aminoferrocene molecules. The spectra of the AFc-GO sheets are characterized by the peaks corresponding to GO ($\upsilon_{\mathrm{C-O}}$ at 1079 cm^{-1}, $\upsilon_{\mathrm{C-O-C}}$ at 1240 cm^{-1}, $\upsilon_{\mathrm{C-OH}}$ at 1401 cm^{-1}, $\upsilon_{\mathrm{C=C}}$ at 1630 cm^{-1}, $\upsilon_{\mathrm{C=O}}$ at 1735 cm^{-1}, $\upsilon_{\mathrm{O-H}}$ at 3387 cm^{-1}) as well as the peaks corresponding to organometallic molecules (ferrocene or aminoferrocene at 816, 1105, 1350, 1447, 3090 cm^{-1}).

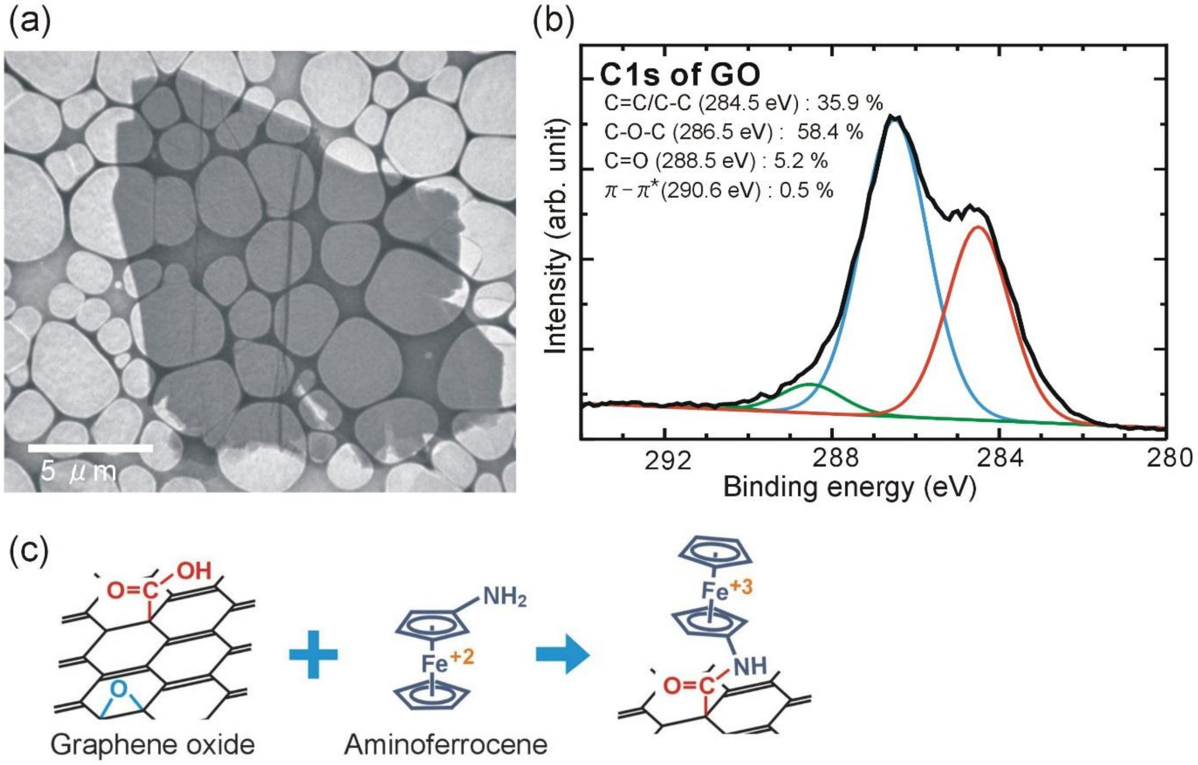

Figure S5. (a) TEM image of a GO sheet. (b) XPS spectra of C1 core level of the GO sheets before the synthesis and fitting curves correspond to components of C=C/C-C (284.5 eV), C-O-C (286.5 eV), and O=C-OH (288.5 eV). (c) Schematic illustration of the coupling reaction between a carboxyl group of a GO sheet and an amino group of an aminoferrocene molecule.

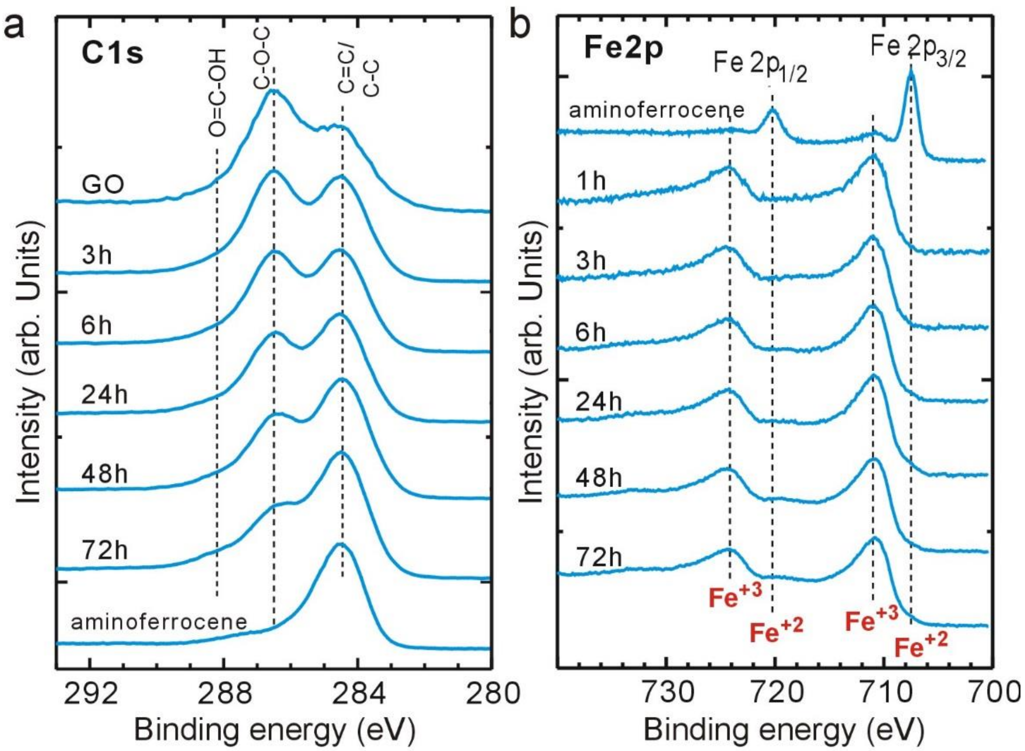

Figure S6. XPS spectra of (a) C1s and b) Fe2p core levels in AFc-GO sheets for each reaction time of the synthesis. In (a), dotted lines show each component. In (b), dotted lines show peaks corresponding to ionized states of Fe.

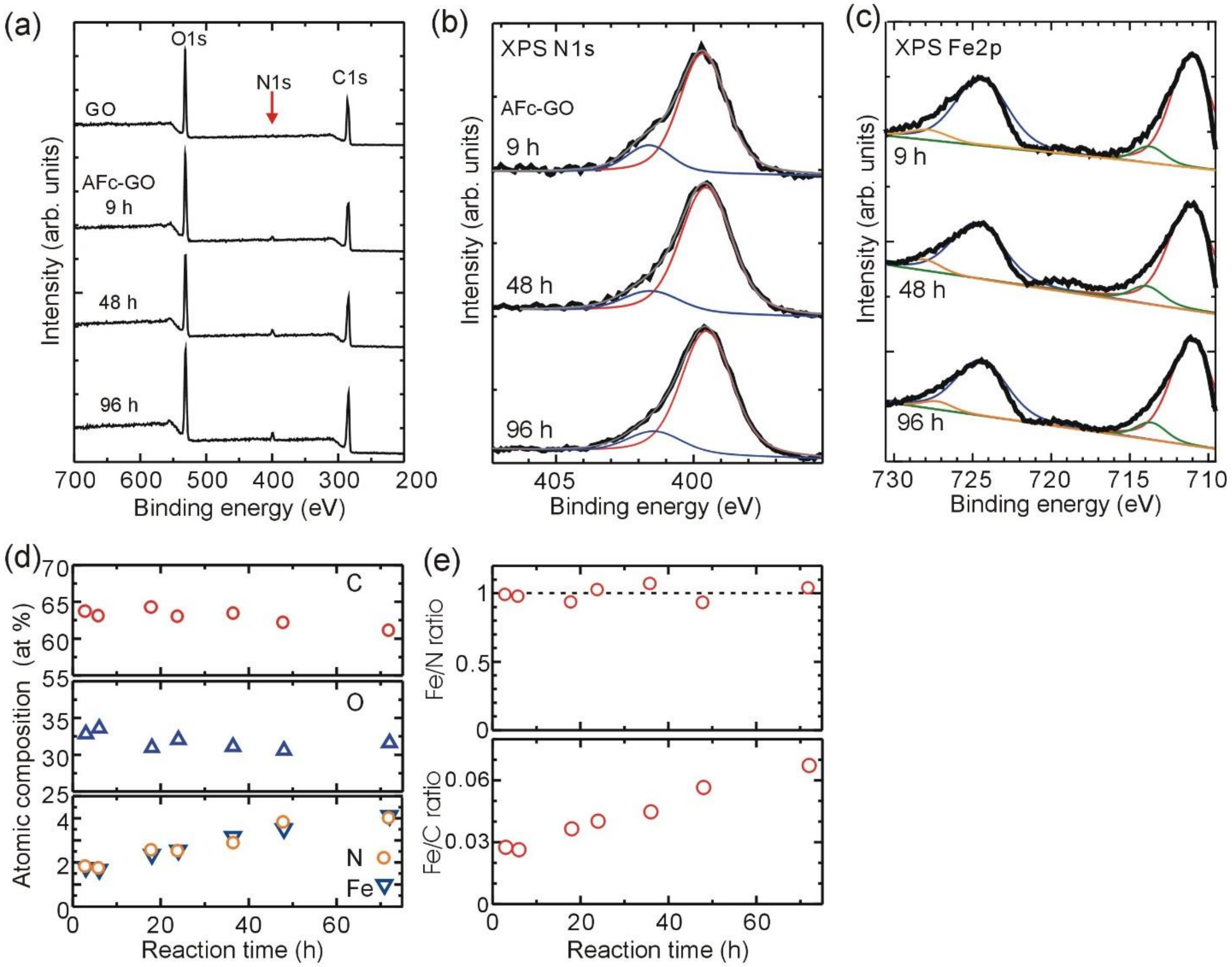

Figure S7. (a) XPS spectra of GO sheets and AFc-GO sheets with reaction time of 9, 48, 96 h. Note that XPS signal of N 1s core level is observed after the synthesis. The de-convoluted spectrum of (b) the N 1s and (c) Fe 2p peaks for the AFc-GO sheets. In (b), peak at 399.7 eV is due to covalent immobilization between the carboxyl groups of the GO sheet and the amino groups of aminoferrocene molecule [4]. In (c), ratios of the Fe^{+2} components drawn by orange and green curves are less than 6 %. (d) Atomic composition obtained from XPS signals of AFc-GO sheets at each reaction time. (e) The Fe/N and Fe/C ratios obtained from XPS signals are plotted against the reaction time. These ratios support the formation of nanoclusters by aminoferrocene molecules.

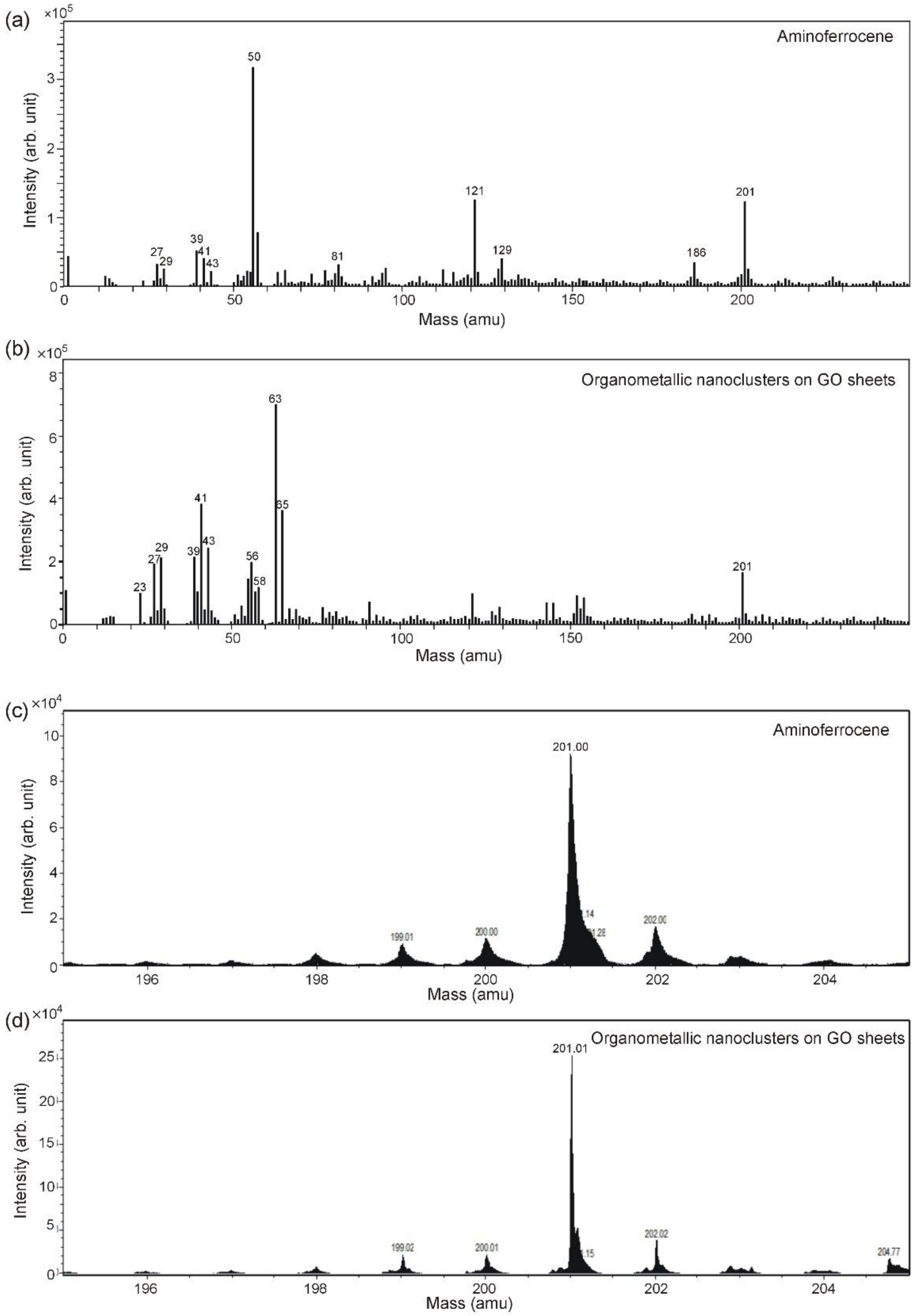

Figure S8. Mass spectroscopy of (a) aminoferrocene powder and (b) AFc-GO sheets (reaction time 72 h) by using TOF-SIMS. (c) , (d) Focused molecular ion peaks of each sample near 201 amu. The peak at 201 amu in both spectra indicates that there are aminoferrocene molecule or positive ionized one. The peaks in the smaller mass regions correspond to fragments of aminoferrocene molecules and GO sheets broken by accelerated ions bombardment.

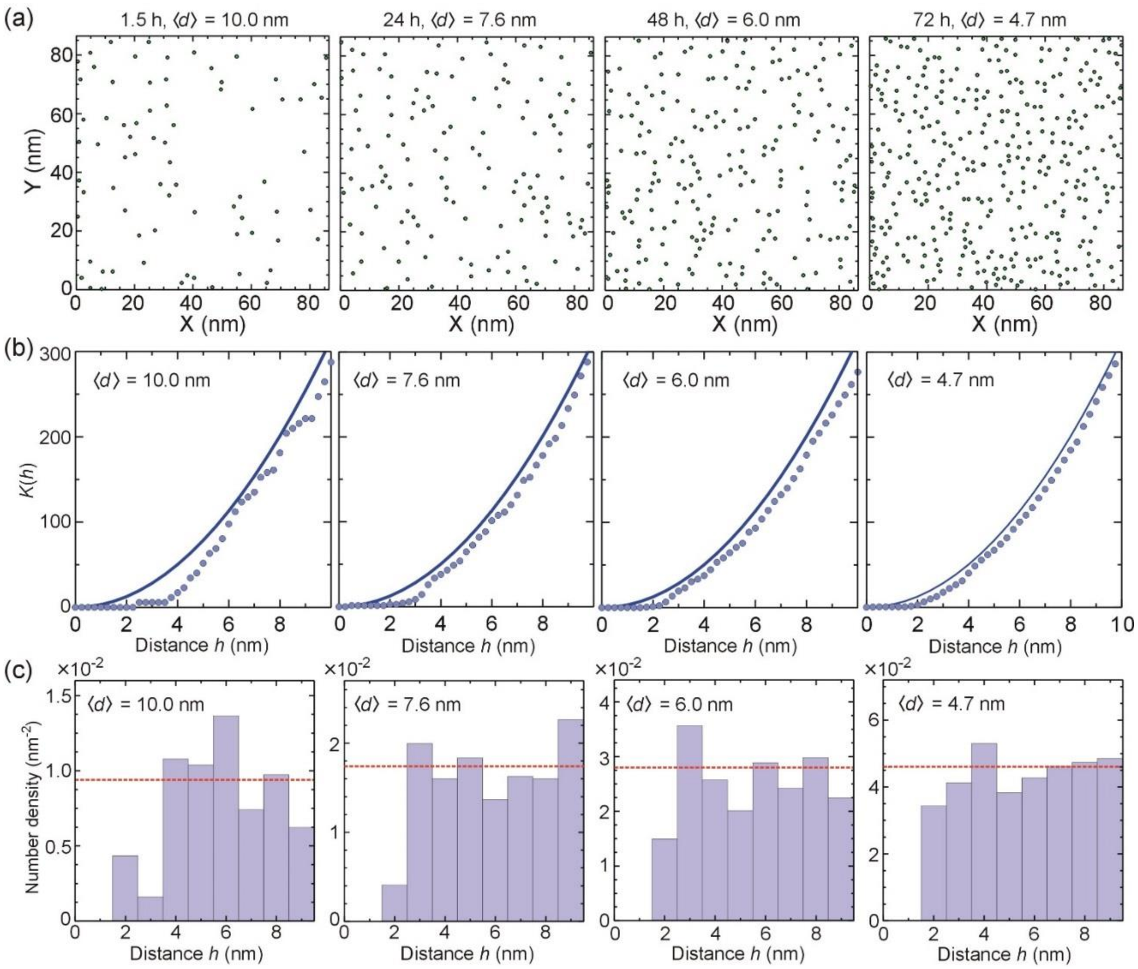

Figure S9. (a) Spatial point patterns corresponding to the positions of nanoclusters for AFc-GO sheets ($\langle d \rangle$ = 10.0, 7.6, 6.0, 4.7 nm: reaction time = 1.5, 24, 48, 72 h). (b) K-function $K(h)$ (Equation (S1) in Supporting Discussion), (c) average number density at distance h from central point for aminoferrocene nanoclusters on GO sheets ($\langle d \rangle$ = 10.0, 7.6, 6.0, 4.7 nm). Each dot in (a) corresponds to the position of the nanocluster. The solid line in (b) corresponds to a uniform distribution. Red dotted lines in (c) show the average number density for each AFc-GO sheet.

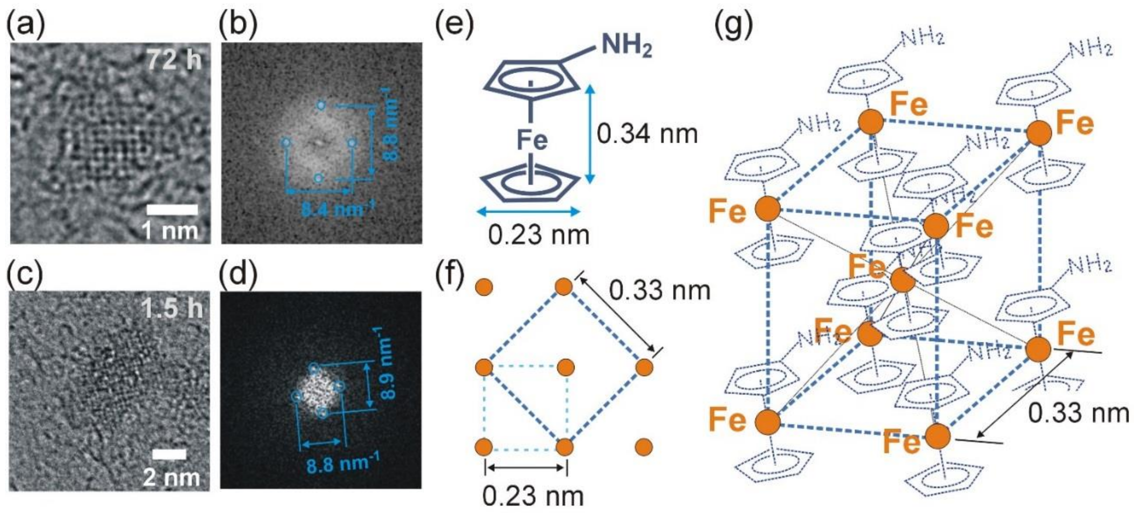

Figure S10. TEM images of aminoferrocene nanoclusters after reaction times of (a) 72 h and (c) 1.5 h, showing a regular arrangement of molecules. Fourier transformed images of each TEM image shows square symmetry in (b) and (d). From the separation of the peaks, the vertical and horizontal periodicity of the nanoclusters are 0.23 ± 0.01 nm and 0.24 ± 0.01 nm for an AFc-GO sheet with the reaction time of 72 h, and 0.23 ± 0.01 nm and 0.23 ± 0.01 nm an AFc-GO sheet with the reaction time of 1.5 h. (e) Schematic molecular structure of aminoferrocene.[3] (f) Top view of the arranged Fe ions in aminoferrocene molecules forming the nanocluster, because the contrast in the TEM image is formed mainly by Fe atoms. (g) Schematic illustration of a face-centered tetragonal structure of the nanocluster. Although information about the lateral arrangement of Fe atoms in the nanoclusters was obtained from the patterns in the TEM image, vertical distance in the arrangement and tilting of the molecules were not estimated from the TEM image. The molecular nanoclusters were stable under electron beam of TEM.

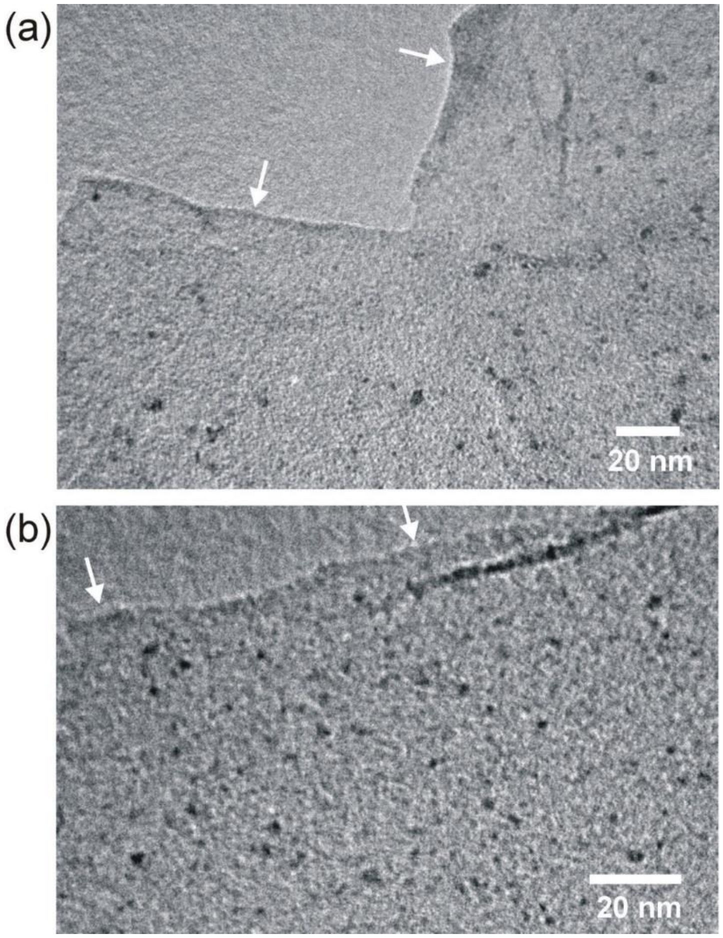

Figure S11. TEM images of AFc-GO sheets with reaction times of (a) 1.5 h and (b) 48 h. In the images acquired using the conventional TEM mode, the nanoclusters correspond to black small dots. White arrows show the edges of the sheet. No nanoclusters were observed at the edge of the AFc-GO sheets.

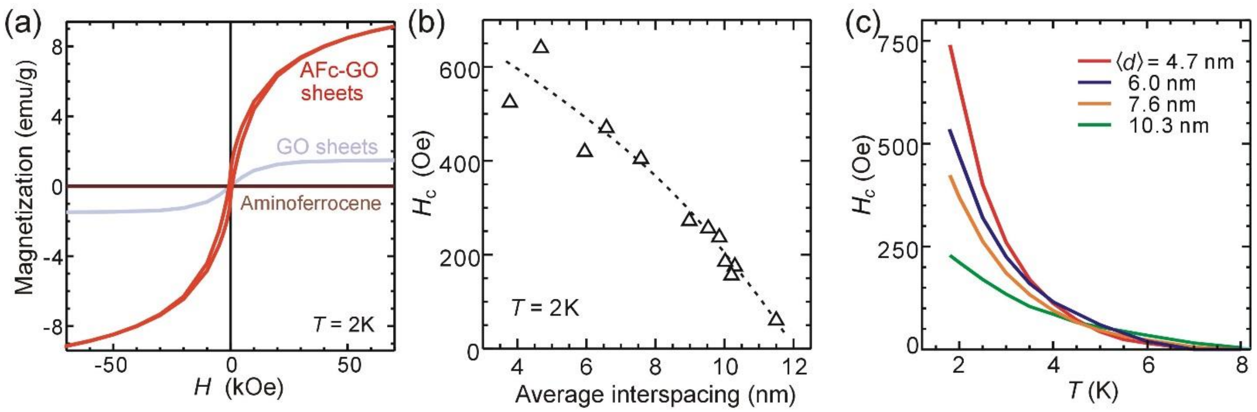

Figure S12. (a) *M*-*H* loops for AFc-GO sheets with reaction time of 72 h, pristine GO sheets, and pristine aminoferrocene powder at $T = 2$ K. (b) Coercive force H_c of AFc-GO sheets at $T = 2$ K against average interspacing between the nanoclusters. The dashed line is a guide to the eye. (c) H_c for AFC-GO sheet ($\langle d \rangle = 4.7, 6.0, 7.6, 10.3$ nm) is plotted against temperature.

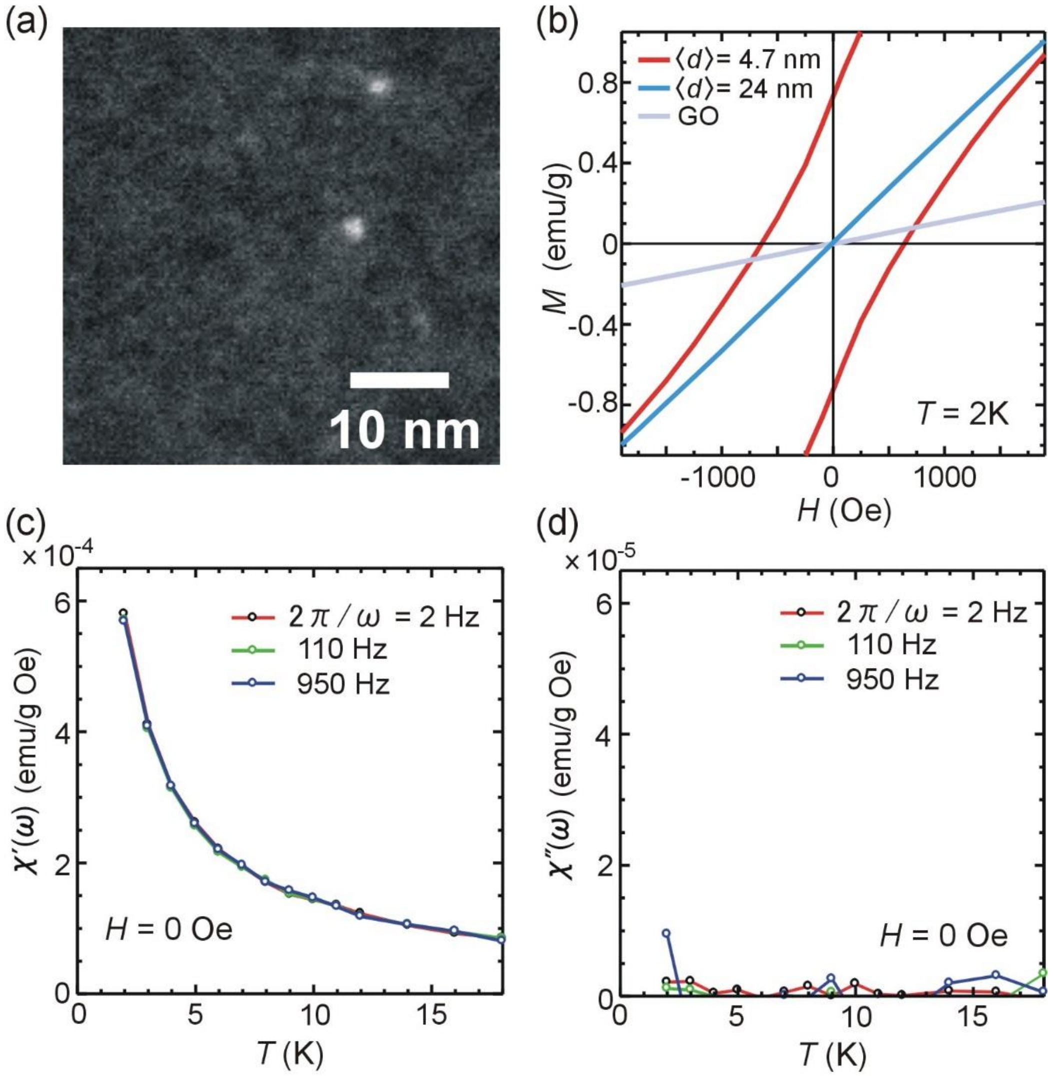

Figure S13. (a) STEM image of the nanoclusters on a GO sheet for AFC-GO sheet ($\langle d \rangle$ = 24 nm). (b) *M*-*H* loop of AFC-GO sheets ($\langle d \rangle$ = 24, 4.7 nm) and GO sheets at T = 2 K. Note that the "almost isolated" nanoclusters ($\langle d \rangle$ = 24 nm) do not show a hysteretic *M*-*H* loop. (c) Real $\chi'(\omega)$ and (d) imaginary $\chi''(\omega)$ components of dynamic susceptibility of AFc-GO sheets ($\langle d \rangle$ = 24 nm, $\omega/2\pi$ = 2, 110, 950 Hz) against temperature.

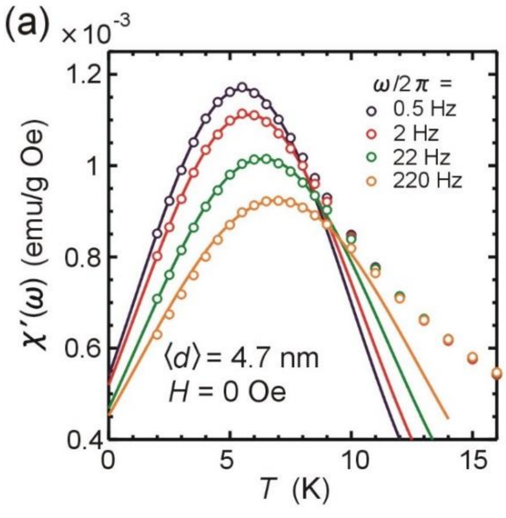

Figure S14. (a) Real $\chi'(\omega)$ components of dynamic susceptibility of AFc-GO sheets ($\langle d \rangle$ = 4.7 nm) as a function of driving frequency ($\omega/2\pi$ = 0.5, 2, 22, 220 Hz) in a zero static field is plotted against temperature (open circle). The susceptibility near the maximum is fitted using a Gaussian function (solid line), giving peak temperature $T_{\mathrm{P}}(\chi')$. (b) Linear relation between $1/T_{\mathrm{P}}(\chi')$ and $\ln(\omega/2\pi)$ in dynamic susceptibility $\chi'(\omega)$ for AFc-GO sheets in a zero static magnetic field.

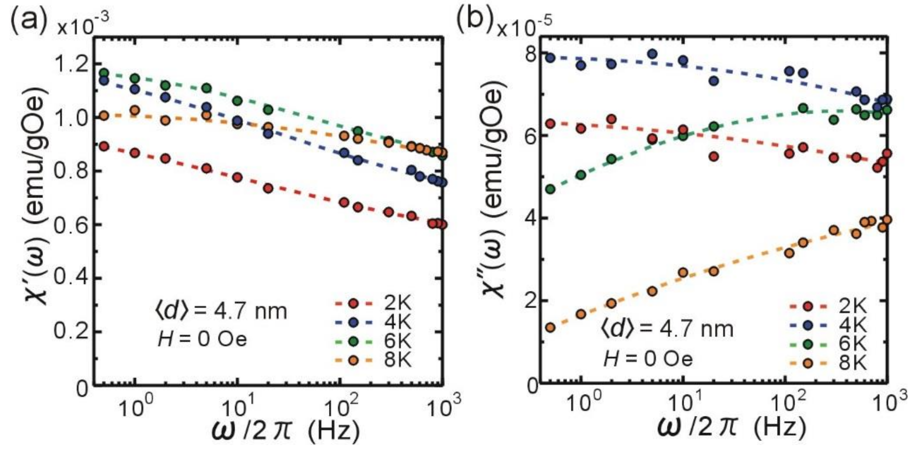

Figure S15. Frequency dependence of (a) the real component $\chi'(\omega)$ and (b) imaginary component $\chi''(\omega)$ of the dynamic susceptibility in a zero static field for AFc-GO sheets ($\langle d \rangle$ = 4.7 nm) at T = 2, 4, 6, 8 K. The dashed line is a guide to the eye.

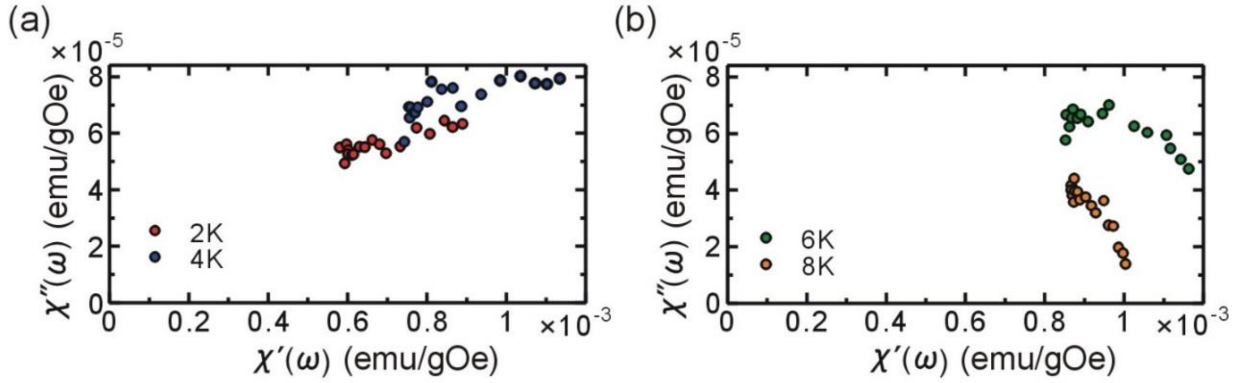

Figure S16. Cole-Cole diagram of dynamic susceptibilities ($\chi'(\omega)$, $\chi''(\omega)$) of AFc-GO sheets ($\langle d \rangle$ = 4.7 nm) at (a) T = 2, 4 K, and (b) 6, 8 K. Note that the vertical scale is about one order smaller than the horizontal scale.

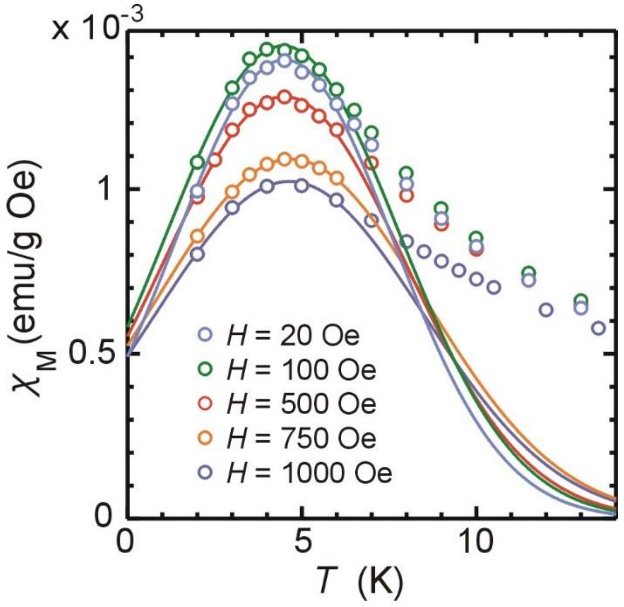

Figure S17. Static susceptibility of AFc-GO sheets ($\langle d \rangle$ = 4.7 nm) under different static field (20 - 1000 Oe). Gaussian fitting is drawn by solid line, giving the peak temperature T_p.

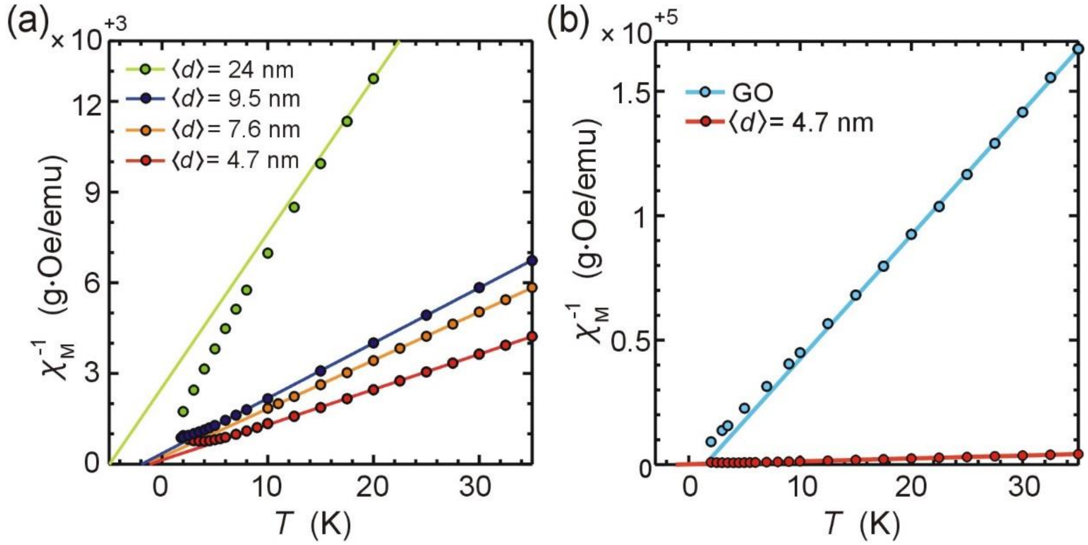

Figure S18. Linear fitting of the inverse of static susceptibility χ_{M} against temperature using the Currie-Weiss law for (a) AFc-GO sheets ($\langle d \rangle$ = 4.7, 7.6, 9.6, 24 nm) and (b) GO sheets and AFc-GO sheets ($\langle d \rangle$ = 4.7 nm).
